# Influence of Reduced Molar Mass of Low-Acyl Gellan Gum on Weak Gel Formation and Rheological Properties

**DOI:** 10.3390/gels11060398

**Published:** 2025-05-27

**Authors:** Nina G. Mikusheva, Ivan M. Zorin, Alexander S. Gubarev, Alexandr V. Ievlev, Olga V. Volina, Nikolai V. Tsvetkov

**Affiliations:** Saint Petersburg State University, 7/9 Universitetskaya Nab., 199034 St. Petersburg, Russiaa.gubarev@spbu.ru (A.S.G.); a.ievlev@spbu.ru (A.V.I.); o.volina@spbu.ru (O.V.V.); n.tsvetkov@spbu.ru (N.V.T.)

**Keywords:** low-acyl gellan gum, gellan, gelling, molar mass, coil–helix transition, ion-induced hydrogel, rheology

## Abstract

Reduced-molar-mass low-acyl gellan gum was obtained by the centrifugation of an aqueous solution of commercially available food-grade gellan gum. The derived sample was characterized by NMR, FTIR, ICPE, and viscometry methods. The characteristics were compared with commercially available gellan gum Gelzan™. The main focus of the investigation is on the rheological properties of low-molar-mass-gellan ion-induced gels and the influence of reduced molar mass on gelling of gellan. The gels were prepared by adding 0.2–0.3 M of NaCl or KCl to the 0.6 g/dL gellan gum aqueous solution in a 1:1 ratio. The kinetics of gelling at room temperature, studied by rheological methods, strongly depends on molar mass and is practically independent of the temperature (up to 37 °C) and the type of ions. Analysis of the mechanical spectra characterized the obtained gels as weak gels. The gelling temperature achieved upon cooling for low-molar-mass gellan with a 0.1 M NaCl concentration was 39.0 °C (rheology) and 43.5–42.5 °C (visual observation). In summary, this study complements the existing knowledge about how the reduced molar mass of low-acyl gellan gum influences its rheological properties and gelling behavior in ion-induced systems and provides insights into the formulation of gellan-based gels, which can be effectively utilized in various food and pharmaceutical applications.

## 1. Introduction

Gellan gum is a natural anionic gel-forming polysaccharide produced commercially by the microbial fermentation from *Sphingomonas elodea* (formerly classified as *Auromonas elodea* [1] or *Pseudomonas elodea* [2,3] (ATCC 31461) [4]. Gellan polymer consists of tetrasaccharide repeating units containing monosaccharides: 1,3-β-D-glucose, 1,4-β-D-glucuronic acid, 1,4-β-D-glucose, and 1,4-α-L-rhamnose in molar ratios of 1:1:1:1 linked together to form a linear primary structure [5]. The native polymer is high-acyl gellan containing O-5-acetyl and O-2-glyceryl (2,3-L-dihydroxypropionyl) groups on the 1,3-β-D-glucose residue. When exposed to alkali at high temperatures, both acyl groups are hydrolyzed, and the deacylated form of low-acyl gellan is obtained.

Gellan molecules may be present as sodium or potassium salts of glucuronic acid; Mg^2^⁺ and Ca^2^⁺ ions can also be performed, depending on the types of ions present in the nutrient salts essential for microbial growth and/or those introduced during post-fermentation processing [6]. At that, dissociation takes place in pure water, and the presence of divalent cations (Mg^2^⁺, Ca^2^⁺) facilitates the formation of both intra- and intermolecular crosslinking.

The ordered structure of gellan in its solid state is a coaxial double helix [7]. In an aqueous solution at room temperature (24–25 °C), gellan molecules adopt a double-helix conformation, turning into a coil conformation with increasing temperature (36–40 °C), which has been confirmed by studies examining the rigidity of the polymer chains and their molar mass [8,9]. At the appropriate concentration, gellan forms thermally reversible gels, which undergo coil–helix transition without visible thermal hysteresis [6]. The higher the concentration of gellan in the solution is, the higher the coil–helix transition temperature is [10].

Within the model of gel formation by gellan in the presence of added cations, proposed in [11], while a heated solution of gellan with added salts is cooled, the cations are embedded between the coils of the resulting spirals, forming crosslinking or shielding charge interactions [12], which leads to the formation of a stronger gel. At the same time, the coil–helix transition temperature increases with the addition of salts [11], accompanying the appearance of a thermal hysteresis [6,13]. The changes in molar mass do not noticeably affect the coil-to-helix temperature while applying Ca^2+^ as a gel-inducing ion [14]. To the best of our knowledge, the influence of molar mass on coil–helix transition temperature during monovalent ion-induced gel formation has not been addressed in the literature.

Gellan gum is a biologically inert polymer [15] with the potential for applications in wound dressing [15,16], tissue engineering [17,18], biosensor synthesis [19], and pharmaceutical formulations [20,21,22,23,24]. Low-molar-mass gellan has barely been investigated in this area; however, it was shown to have several distinctive qualities, particularly concerning its antioxidant capacity [25] and impact on plant growth [26]. To a greater extent, the interest in low-molar-mass gellan can be attributed to its unique rheological properties, and some of them are addressed in this paper.

In situ hydrogels refer to the polymeric solutions which undergo sol–gel phase transition, resulting in a viscoelastic gel in response to specific stimuli [27,28]. This gelation process can be initiated by variations in temperature, pH levels, or ionic concentrations, and it can also be stimulated through UV light exposure. For drug delivery applications, these polymers are combined with therapeutic agents while in a liquid state; upon administration, the solution creates an in situ gel depot at the physiological temperature [29]. In the context of ocular applications, certain ion-activated systems are formulated using a combination of gellan and chitosan [29], or gellan and hydroxyethylcellulose [30]. At the same time, multiple research studies have been focused on the development of thermosensitive gels which respond to changes in temperature [31,32]. Several thermogelling polymers have been reported for ocular drug delivery, including poloxamers, multiblock copolymers made of polycaprolactone, polyethylene glycol, poly (lactide), poly (glycolide), poly (Nisopropylacrylamide), and chitosan [31]. However, the coil–helix transition temperature for the majority of gellan gels typically exceeds the physiological temperature range.

This article presents the results on investigation of a weak gel formation based on low-molar-mass gellan, formed at temperatures between 25 and 37 °C without the need for any supplementary thermal exposure. This gelling process occurs when a solution of low-molar-mass gellan is mixed with a sodium or potassium salt solution, resulting in a gelling time that is shorter than that of high-molar-mass gellan. Additionally, the article examines the mechanical spectra, offering insights into the additional properties of these systems that may be beneficial in the context of ocular drug delivery applications. Furthermore, the thermal gelling behavior upon cooling was studied by rheological methods and visual observation.

The authors believe that the ability of low-molar-mass gellan to modify temperature characteristics and gel consistency allows for innovative formulations tailored to meet specific application needs. The main focus of the present study is on the rheological properties of low-molar-mass-gellan ion-induced gels and the influence of reducing molar mass on gelling of gellan.

## 2. Results and Discussion

Food-grade gellan Caramella purchased from Craftology (Russia) was left to swell in deionized water for 1 h. The obtained “semi-solution” was centrifuged at 6000 rpm for 80 min, and the gel fraction was separated. The separated solution was subsequently dialyzed against water and after 1 day, the membrane solution was centrifuged at 6000 rpm for 40 min and dried lyophilically. This resulted in obtaining dry, fluffy, white fibers marked as Gellan-1; the final yield was 32%.

On the other hand, gellan gum purchased from Merch (Gelzan™) was taken as a standard of a laboratory-grade gellan gum and used for the comparison. This sample was marked as Gellan-2.

### 2.1. Characterization of Gellan Samples

#### 2.1.1. Inductively Coupled Plasma Atomic Emission (ICPE) Analysis

The cations containing in the gellan can facilitate gelling and form inter- and intramolecular crosslinks (Ca^2+^, Mg^2+^). The cation content of gellan samples is therefore crucial for gel characteristics. Cation content, namely Na^+^, K^+^, Ca^2+^, and Mg^2+^, of the used gellan samples is listed in the Table 1. These data indicate that during the processing of the food-grade gellan, the ion content was reduced accordingly. The content of the different cations in Gellan-1 and Gellan-2 was the same by the order of magnitude. The difference in the content of Ca^2+^ and Mg^2+^ (the most efficient facilitators [6]) together between Gellan-1 and Gellan-2 was within the experimental error.

#### 2.1.2. Nuclear Magnetic Resonance (NMR) Analysis

The content of acyl groups significantly affects the mechanical properties of the obtained gel. The gels vary from soft and elastic for the acylated form to hard and brittle for the fully deacylated gellan gum [11,33].

**Figure 1:**
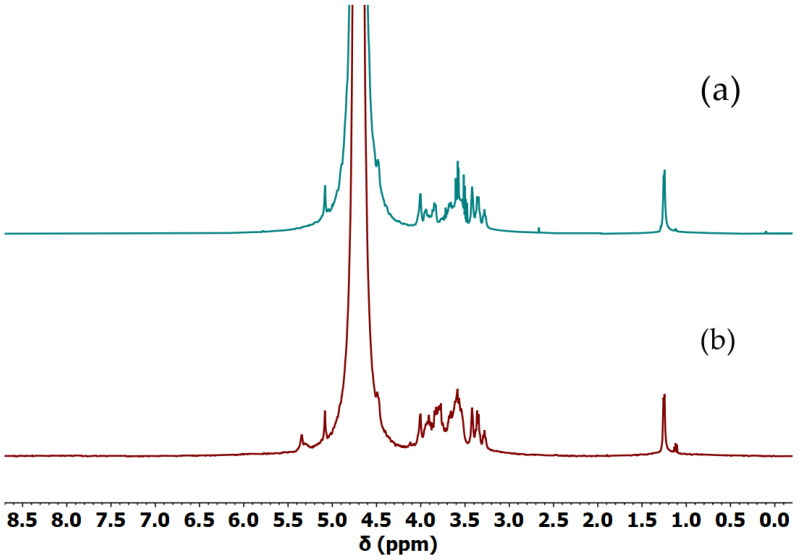
The ^1^H NMR spectra of (**a**) Gellan-1 in D_2_O, (**b**) Gellan-2 in D_2_O.

In order to determine whether our gellan samples were acylated or not, the ^1^H NMR spectrum of acylated and deacylated gellan was calculated and used as reference (Figure S1). The simulation clearly showed the position of the signal from the additional methyl group around 2 ppm. The comparison of spectra of the food-grade gellan gum used for purification, the calculated acyl gellan gum spectra, and the obtained Gellan-1 revealed that the part containing acyl groups was completely removed during the preparation of the sample (Figure S2). The spectra of Gellan-1 and Gellan-2 are shown in Figure 1.

It can be concluded that Gellan-1 was a deacylated gellan gum sample, which corresponded to the Gellan-2 in the NMR spectrum. Also, NMR spectra showed the presence of low-molar-mass impurities, which did not contradict the ICPE data.

#### 2.1.3. Fourier Transform Infrared (FTIR) Spectroscopy

**Figure 2:**
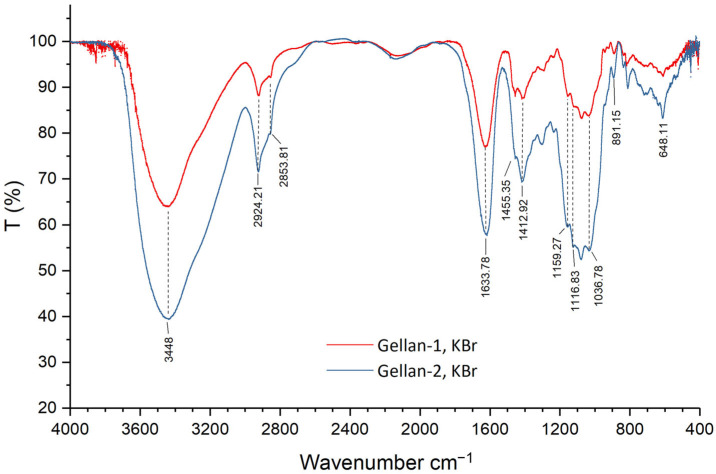
FTIR spectra of Gellan-1 and Gellan-2.

The FTIR spectra of Gellan-1 and Gellan-2 are shown in Figure 2.

The spectra of Gellan-1 and Gellan-2 (Figure 2) were practically identical and had a characteristic peak at 3448 cm^−1^ (hydroxyl O-H stretching). The peak around 2924 cm^−1^ (C-H_2_ asymmetric stretching band) [33] almost overlapped with the peak at 2854 cm^−1^ (CH_2_ symmetric stretching band [34,35]), which was rather small compared to acylated gellan (Figure S3). The peaks at 1634 and 1413 cm^−1^ were connected with the presence of asymmetric and symmetric COO– stretching groups, correspondingly. The small shoulder at 1455 cm^−1^ could be assigned to the “scissoring” vibrations in the CH_2_ groups. The peak at 1159 cm^−1^ corresponded to C-O stretching vibration. The spectrum of Gellan-1 had no peak in the wavenumber region of 1750–1680 cm^−1^ (1745 cm^−1^), which refers to the stretching vibration of C=O bound in the acyl groups [35,36,37]. Together with a relatively small peak at 2854 cm^−1^ (CH_2_ symmetric stretching band), this indicates that the part containing acyl substituents was removed during the preparation of the sample Gellan-1. This result correlates with the NMR data.

#### 2.1.4. Viscometry

The intrinsic viscosity [η] is the most sensitive hydrodynamic parameter to the size and conformation of a polymer chain in a solution. The intrinsic viscosity and molar mass M are related by the Mark–Kuhn–Houwink–Sakurada empirical ratio:

[η] = K · M^a^ (1)

While examining the viscosity of dilute solution containing individual molecules of a polymer, the value of the intrinsic viscosity can be obtained using the Huggins [38] and Kraemer [39] extrapolation procedures. The values of the Huggins and Kraemer constants are related to the thermodynamical quality of the solvent [40]. The internal consistency of the viscometry data can be verified by simultaneous implementing the Huggins and Kraemer plots using standard dilution procedures [41]; both of them should be approximated by straight lines, and both equations should result in the same value of the characteristic viscosity, unless the thermodynamic quality of a solution worsens [42].

Due to the electrostatic repulsion of the charged groups bared by the polyelectrolyte chains, the reduced viscosity of polyelectrolyte solutions increases as the local concentration of the counterions becomes lower, which is different from the viscosity behavior usually observed for neutral polymers. This effect is known as the “polyelectrolyte effect” [43,44,45,46]. The addition of salts, or, rather, counterions, to a polyelectrolyte solution reduces electrostatic repulsion by screening the charge interactions. In this case, the polyelectrolyte takes a more compact conformation, which results in the decrease of macromolecular coil volume and the determination of values of the reduced viscosity becomes feasible. It is common practice to use NaCl or KCl solutions of the appropriate concentration as a solvent.

**Figure 3:**
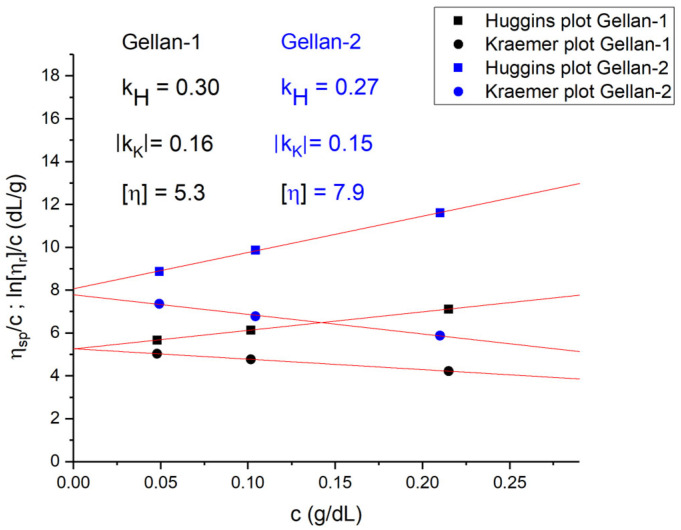
The normalized specific viscosity, η_sp_/c (Huggins plot) and ln(η_r_)/c (Kraemer plot) on polymer concentration, c (g/dL) at 25 °C. k_H_ and k_K_ are the Huggins and Kraemer constants, correspondingly.

Considering gellan, it is important to note that aggregation (gelation in more concentrated solutions) occurs upon the addition of sodium or potassium salts [47]. This prevents the determination of molar mass by hydrodynamic methods. However, if we can identify a solvent that effectively suppresses the polyelectrolyte effect while the salt concentration remains below the threshold necessary for aggregate formation, such an analysis becomes feasible. Based on the ICPE data (Table 1) providing information on the ion content in the gellan samples, the solvents containing the double concentration of Na^+^, K^+^, and Ca^2+^ were prepared, namely (0.0052M KCl + 0.0014M NaCl + 0.0010M CaCl_2_) for the dilution of the 0.43 g/dL aqueous solution of Gellan-2 and (0.0034M KCl + 0.0020M NaCl + 0.0006M CaCl_2_) for the 0.43 g/dL solution of Gellan-1. Further standard solution procedures were implemented. The Huggins and Kraemer plots were approximated by straight lines; the values of the Huggins and Kraemer constants corresponded to the values characteristic for flexible chain polymers in thermodynamically good solvents (Figure 3) [40,41,48]. Additionally, the known interrelation of the Huggins and Kraemer parameters k_H_ + |k_K_| = 0.5 was almost fully satisfied. The obtained values were [η]_Gellan-1_ = 5.3 dL/g; [η]_Gellan-2_ = 7.9 dL/g.

The intrinsic viscosity of Gellan-1 was lower than intrinsic viscosity of Gellan-2, revealing that Gellan-1 had a lower molar mass than Gellan-2.

#### 2.1.5. Determination of Molar Mass

The exponent degree indicator of Mark–Kuhn–Houwink–Sakurada in good and theta solvents varies from 0.5 for Gaussian coil conformation up to 1.8 for a polymer chain in a rigid stick conformation. Dreveton et al., in 1996 [47], determined the Mark–Kuhn–Houwink relationship for gellan gum in 0.025M TMACl as [η] ∝ M^0.92^ (calculated for the single-chain conformation). Takahashi et al., in 2004 [9], determined the Mark–Kuhn–Houwink relationship for gellan gum in 0.025M NaCl at 40 °C (the single-chain conformation) as [η] ∝ M^0.88^. In the general case, the exponent a in the KMH equations for viscosity is 0.5 under ideal conditions. Thus, the increased value of 0.9 (average of the reported data) may refer to either a rigid-chain conformation (polyelectrolyte stiffness due to low salt concentrations or structural peculiarities) affecting the manifestation of draining effects, or a condition of thermodynamically good solvent [49]. Since the constant a (degree indicator in Equation (1)) did not change dramatically while staying in thermodynamically good conditions, this value was taken to compare the Gellan-1 and Gellan-2 molar masses in the first approximation. Considering Gellan-1 and Gellan-2 being polymer homologues, the Equation (1) combined with the determined value of degree indicator leads to the equation

[η] _Gellan-2/_[η] _Gellan-1_ = (M _Gellan-2_ ⁄ M _Gellan-1_)^0.9^, (2)

Thus, the molar mass of Gellan-1 was 1.6 times smaller than molar mass of Gellan-2. This result correlates with the analytical ultracentrifugation (AUC) data (Table 2).

The molar mass M_sf_ can be calculated via equation

$M_{sf}=9\pi \sqrt{2}N_{A}{\left(\left[s\right]f/f_{0}\right)}^{3/2}\sqrt{\upsilon }$, where $[s]=\frac{s_{0}\eta_{0}}{\left(1-\upsilon \rho_{0}\right)}$ is the intrinsic sedimentation coefficient, N_A_ is Avogadro’s number, and υ is the partial specific volume.

The consistency of the obtained data can be checked via hydrodynamic invariant A_0_, combining values of solvent viscosity η_0_, diffusion coefficient D_0_, molar mass M, intrinsic viscosity [η], and temperature T: $A_{0}=\eta_{0}D_{0}{\left(M[\eta ]\right)}^{1/3}/T$.

The obtained A_0_ values are typical for polymer chains in thermodynamically good solvent. But it should be emphasized that the obtained AUC data are evaluative in nature. Firstly, the measurements were conducted at a single concentration. Secondly, due to the inhibitive effect of counter-ions, the sedimentation rate of the polymer chains decreased, leading to what could be described as an “apparent” sedimentation coefficient, which might be lower than the true one. This could result in an underestimation of the molar mass. However, comparison based on masses remain relevant, and the significantly high value of the frictional ratio—indicative of greater asymmetry in the sedimenting particles—aligned well with the expected high equilibrium rigidity.

According to the Mark–Kuhn–Houwink equation for gellan [47], the molar mass can be estimated as following: M_Gellan-1_ = 110 kDa, M_Gellan-2_ = 165 kDa.

**Figure 4:**
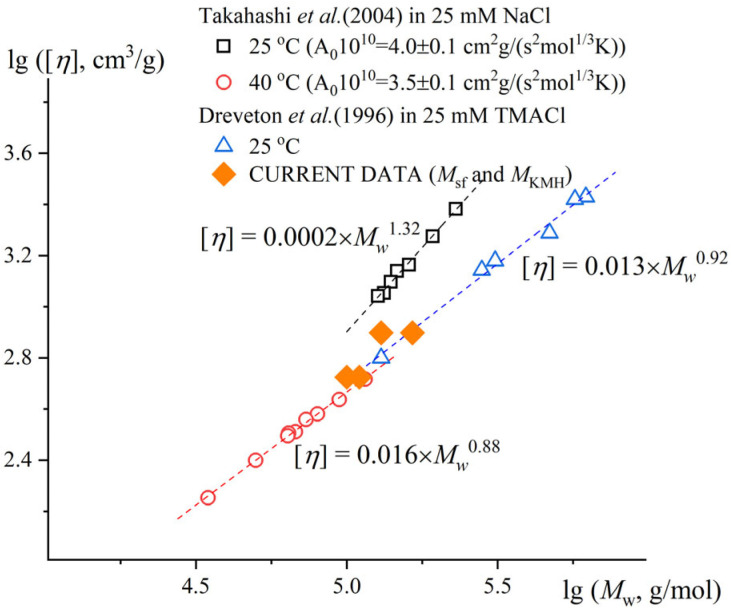
The Mark–Kuhn–Houwink extrapolation, which integrates the estimated molar masses from the current study (orange) alongside literature data [9,47].

The molar mass values estimated generally correlated well with the existing literature data, and the generalizing illustration is shown in Figure 4.

The calculated values of hydrodynamic invariant A_0_ for [9] were A_0_ = 4.0 ×⋅10^−10^ for the more rigid conformation of the double helix and 3.5⋅× 10^−10^ g⋅cm^2^/(s^2^K mol^1/3^) for the monomolecular conformation, which align well with the concept of hydrodynamic invariant [49].

#### 2.1.6. Partial Specific Volume

The partial specific volume of a solute is a characteristic parameter that can be used in studies on solvent interactions and other intermolecular interactions [50] and should not depend on molar mass while considering polymer homologues series. The partial specific volume of Gellan-1 determined in aqueous solution was 0.66 cm^3^/g, and of Gellan-2 was 0.61 cm^3^/g (Figure S4); these were in satisfactory agreement with each other.

### 2.2. Influence of Molar Mass on Gelling Kinetics

It has been observed that when 0.2M NaCl aqueous solution is added to an aqueous gellan solution at a concentration of 0.6 g/dL in a 1:1 ratio at room temperature, gelling occurs throughout the entire volume. Gellan with a high molar mass requires a significant amount of time to gel, while gellan with a lower molar mass gelled much more quickly.

The gelling kinetics were studied using coaxial cylinder geometry (CC-10) with a Peltier temperature control device. To minimize the impact of the measuring method on gelling, a shear strain of 0.1% and a frequency of 1 rad/s were applied.

The gellan solutions with a concentration of 0.6 g/dL, the 0.2M NaCl solution, and the measuring cell were thermostatted at 25 °C. Then, 0.5 mL of the gellan solution was poured into the measuring cell, the solution was additionally thermostatted; the measuring geometry was lowered to the level of 27 mm, then 0.5 mL of 0.2M NaCl aqueous solution was added to the measuring cell, and within 30 s, the measuring system was lowered to the measuring position.

**Figure 5:**
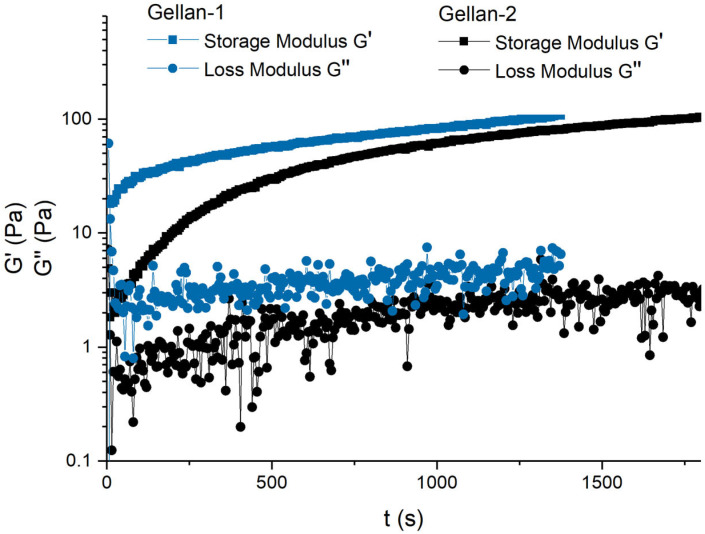
Storage (G′, squares) and loss (G′′, circles) moduli as a function of time (t) for the Gellan-1 (blue) and Gellan-2 (black) aqueous gels; gellan concentration was 0.3 g/dL, and NaCl content was 0.1M.

Thus, the measurement of the loss and storage modules began 30 s after the actual mixing of the sample solution and the salt solution. The measurement time was chosen sufficient to quantitatively confirm differences in the gelling kinetics. Figure 5 shows that the gel with the Gellan-1 sample reached high elastic modulus values faster than the Gellan-2 sample. To be more precise, 85% of the maximum value of the storage modulus (G′_max_) was achieved one and a half times faster in Gellan-1 than in Gellan-2, and 20% of the G′_max_ in Gellan-1 was achieved after 30 s of measurement, while Gellan-2 required more than 6 min. However, there was no significant difference in the absolute values of the final storage modulus value.

In the absence of exposure, gels formed faster, but such measurements showing the dynamics of the increase in mechanical properties are not possible.

The observed dependence can be explained as follows: when an aqueous solution of gellan is mixed with a solution of monovalent salt at room temperature, gellan remains in a helical conformation. This configuration complicates the incorporation of sodium or potassium ions into the helices (considering the model proposed by Robinson [11]).

However, due to the low concentration of gellan, the number of intersections between the helixes was rather small, resulting in a relatively loose grid, even for gellan of higher molar mass. This allows for the preparation of gel from gellan with the addition of monovalent salt, even without the need for additional exposure to temperature. Then, when we consider gellan molecules with lower molar mass and, consequently, shorter contour chain lengths, the mobility of molecules and looseness of the grid increases. In such a system, the mixing of salt and gellan molecules is easier, which explains the faster formation of the gel. The conclusion about the decrease in the number of intersections and the disappearance of the grid in the gellan solution with a decrease in the molar mass of gellan is also consistent with the result obtained earlier by other scientists [25]. In the mentioned work, no gel occurred from gellan of a lower molar mass when the heated solution was cooled, while the “large” gellan under the same conditions resulted in a classical gel.

An increase in temperature to 37 °C did not have a noticeable effect on the rate of gel formation (Figures S5 and S6), nor did the replacement of NaCl with KCl (Figure S7); however, the value of the storage modulus increased. The increase in the values of storage modulus of the gel caused by group I cations in the Na-K-Cs line is a well-known fact [6].

Thus, the high rate of gel formation when mixed at both room and human body temperature makes it possible to consider low-molar-mass gellan as a suitable base for formulations that gel directly after application.

The obtained values of the storage modulus were of the same order of magnitude as those obtained for gels from the pure low-acyl gellan solution under cooling, within a gellan concentration range 0.75–2 g/dL [10,51]; an order of magnitude smaller than those for hydrogels proposed for medical applications, such as wound dressings [15,52]; and two orders of magnitude less than those for gels made from gellan without the addition of salt, even in cases where gels were prepared from low-molar-mass low-acyl gellan, with a concentration of 5 g/dL [14].

### 2.3. Frequency Dependence Analysis

Dynamic viscoelastic properties of the obtained gels (Table 3) were studied using coaxial cylinders geometry, at a shear strain 0.3% (below LVR limit) at 25 °C. The gellan concentration in all cases was 0.3 g/dL. G1 and G4 differed in the molar mass of gellan (Gellan-1 and Gellan-2), and G1–G3 differed in the inducing ion type and ion concentration. In all cases, gelling occurred in the entire volume of the sample.

**Figure 6:**
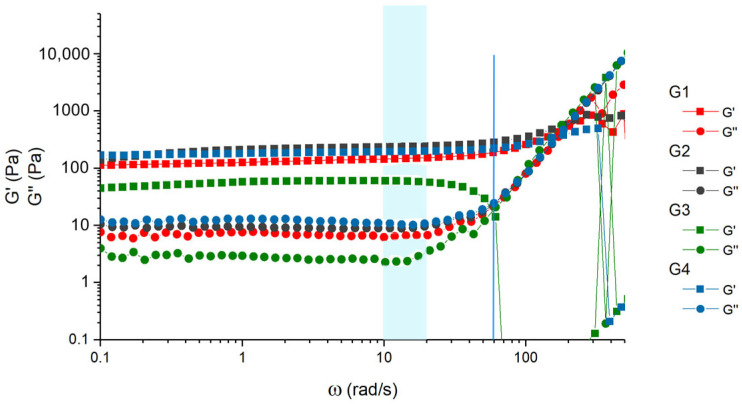
Frequency dependencies of G′ and G′′ for G1–G4 in the angular frequency (ω) range of 0.1–628 rad/s at 25 °C.

The mechanical spectra are presented in Figure 6.

For all gels, the storage modulus (in the linear region) was about two orders of magnitude higher than loss modulus, with both moduli being largely independent of frequency, which is a sign of a gel, not a polymer solution [53]. At the same time the linear viscoelastic strain was below 1% (Figure S8), which qualified these gels as “weak” [53], as well as the values of the loss factor (tan δ = G″/G′) (Figure S9).

The frequency dependence changed at an angular frequency higher than 10–20 rad/s, and both the storage and loss moduli became more strongly frequency-dependent, both increasing with angular frequency. Such dependence is common for hydrogels [15,52]. The G3 gel with the excess of KCl (0.15M) underwent gel–sol transition at 60 rad/s, and the crossover point is marked in Figure 6. Together with the decrease in the value of the storage modulus, this indicates the weakening of the gel.

The maximum absolute values of storage moduli in the linear region were reached at approximately 10 rad/s and are listed in Table 3 (G′_max_). The highest value of G′_max_ was obtained for the gel containing 0.10M KCl, while the lowest gel–sol transition frequency was observed for the gel containing 0.15M KCl (60 rad/s, Figure 6). We believe that establishing the optimal balance between mechanical properties through the variation of added salt can optimize the formulation to meet specific application needs.

### 2.4. Thermal Behaviour

The thermal behavior of low-molar-mass gellan in the presence of sodium salt (0.1M NaCl) was studied using a parallel plate geometry (50 mm, 1 mm gap). Loss (G″) and storage (G′) moduli were measured upon cooling from 90 to 25 °C with a cooling rate 3 °C/min, at a frequency of 1 Hz, and a shear strain of 0.3% (below the LVR limit).

**Figure 7:**
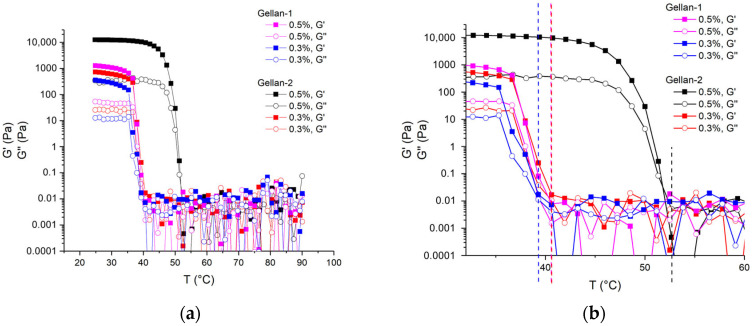
(**a**) Temperature (T) dependence of loss (G″, open) and storage (G′, filled) moduli upon cooling; (**b**) the same dependence more closely illustrating the coil–helix transition temperature (T_ch_). Te concentration in % corresponds to g/dL. The obtained temperatures were: T_ch_^Gellan-2, 0.5%^ = 52.7 °C (black), T_ch_^Gellan-2, 0.3%^ = 40.5 °C (red), T_ch_^Gellan-1, 0.5%^= 40.5 °C (magenta), T_ch_^Gellan-1, 0.3%^ = 39.0 °C (blue).

The coil-to-helix transition results in a rapid increase of the storage modulus value, and the coil-to-helix transition temperature (T_ch_) can be determined as the start of the steep increase in G′ on cooling [6]. The obtained dependencies and T_ch_ are shown in Figure 7a,b.

For gellan alone, the coil-to-helix transition temperature decreases along with the decrease of gellan concentration [10]. It is also known that the addition of salt increases the coil-to-helix transition temperature [6,11]. At the same time, the changes in molar mass do not affect noticeably the coil-to-helix temperature while applying Ca^2+^ as a gel-inducing ion [14].

The obtained dependences illustrate that with a decrease in the concentration of gellan, T_ch_ decreased significantly for high-molecular Gellan-2, while the same change in T_ch_ for low-molecular Gellan-1 was less pronounced. With a decrease in the molar mass, T_ch_ decreased, and this change was more pronounced in the region of a higher concentration of gellan (0.5 g/dL), while at a gellan content 0.3 g/dL, it was barely distinguishable. The obtained value 39.0 °C for the 0.3% Gellan-1 solution in 0.1M NaCl was very close to the normal temperature of human body, and the observed decrease in the transition temperature due to a decrease in molar mass could be considered beneficial for practical use.

At the same time, the presence of mechanical exposure and cooling can lower the observed transition temperature from a coil to a helix, resulting in changes in the gelling behavior. To evaluate this, a gel composed of Gellan-1 at a concentration of 0.3 g/dL with 0.1M NaCl was heated and then allowed to cool gradually. A thermostatted setup was created using a glass of water sealed with a foam lid, with a glass cup containing the gel and a thermometer mounted into it. During the slow cooling process, the temperature of all components was considered the same. The state of the gel/solution was then assessed by short lifting the entire setup and evaluating the texture organoleptically: whether it was still liquid or had thickened. It was found that gelling occurred between 43.5 and 42.5 °C.

Notably, this low-molecular-gellan-based gel can endure multiple heating cycles, and when exposed to temperatures between 70 and 80 °C, it rapidly transforms into a homogeneous mixture, in contrast to gel formed by high-molar-mass gellan. It can be assumed that in the case of shorter molecules, the untangling of the spirals is facilitated, which explains the easier transition from the gel-to-sol state (helix-to-coil transition) during heating.

## 3. Conclusions

The reduced-molar-mass low-acyl gellan gum (referred to as Gellan-1) was obtained through the centrifugation of an aqueous solution of commercially available food-grade gellan gum. The gellan gum sourced from Merck (Gelzan™), termed Gellan-2, served as a standard for laboratory-grade gellan and was used for comparative analysis. The molar masses of Gellan-1 and Gellan-2 were compared by viscosity measurements, and Gellan-1’s molar mass was 1.6 times smaller than the molar mass of Gellan-2. The comparative study showed no significant differences in ion content or density between the two samples, and the NMR and FTIR spectra exhibited good correspondence. Therefore, these two samples were selected for a comparative analysis of the influence of reduced molar mass on gelling properties.

The comparative study of gelling kinetics demonstrated that the low-molar-mass gellan-based gel, induced by monovalent ions, reaches high elastic modulus values significantly faster than the native gellan (Gellan-2) at both room temperature and normal body temperature (25 °C and 37 °C, respectively). This rapid gel formation under both conditions suggests that low-molar-mass gellan could serve as an effective base for formulations intended to gel immediately upon application.

The gels formed without the application of heat were characterized through frequency dependence analysis and classified as weak gels. Notably, the maximum values of storage moduli for both low- and high-molar-mass gellan were found to be of the same order of magnitude within the concentration range examined. 

Furthermore, the coil-to-helix transition temperature of gels produced through heating and subsequent cooling (from 90 °C to 25 °C) was observed to decrease with a reduction in molar mass for the gellan concentrations ranging from 0.3 to 0.5 g/dL. In the rheological studies, this transition temperature was found to reach 39 °C for 0.3 g/dL of low-molar-mass gellan. This temperature reduction may have potential utility in formulations requiring specific thermal responsiveness.

## 4. Materials and Methods

### 4.1. Materials

Food-grade commercial low-acyl gellan Caramella was purchased from Craftology (Craftology, Perm, Russia). Food-/reagent-grade low-acyl gellan Gelzan™ CM (1000 kDa) and sodium chloride (NaCl, 58.44 Da, purity ≥ 99.0%) were obtained from Sigma-Aldrich (Merck KGaA, Darmstadt, Germany). Potassium chloride (KCl) 3 mol/L aqueous solution was received from Mettler Toledo (Mettler Toledo, Nänikon, Switzerland). Calcium chloride (CaCl_2_) 100 mg/mL aqueous solution was obtained from Slavyanskaya Apteka (Slavyanskaya Apteka, Moscow, Russia) (purity ≥ 95% in dry matter). The gellan gum and NaCl aqueous solutions were prepared using ultrapure (Type 1) water obtained using the Direct-Q^®^ 8 UV Water Purification System (Merck KGaA, Darmstadt, Germany). Deuterium oxide (D_2_O) was purchased from Chemical Line (Chemical Line, Saint Petersburg, Russia). All reagents were used as received.

### 4.2. Preparation of Low-Molar-Mass Low-Acyl Gellan Gum

In total, 761 mg of food-grade gellan (Caramella) was left to swell in 61 mL of deionized water for 1 h. The resulting “solution” was centrifuged at 6000 rpm for 80 min using the laboratory centrifuge, and the gel fraction was separated. The separated solution was placed in a MWCO (molecular weight cut-off) 12,000 dialysis membrane. Dialysis was performed against 600 mL of water, and the water was changed once after 2 h. After 1 day, the membrane solution was centrifuged at 6000 rpm for 40 min and freeze-dried (the solution was frozen in liquid nitrogen for at least 10 min using a round-bottom flask, then attached to the vacuum manifold of a freeze dryer (Zirbus VaCo 2, condenser temperature −80 °C) and dried under a low pressure (<25 Pa) until a constant mass was achieved). The final yield was 244 mg (32%).

The obtained sample (Gellan-1) was characterized by NMR, ICPE, and viscometry methods.

### 4.3. Methods

#### 4.3.1. Nuclear Magnetic Resonance (NMR) Spectroscopy

^1^H-NMR spectra were recorded by a Bruker Avance III 500 MHz spectrometer in D_2_O at T = 25 °C. To record the spectra, a standard zg30 pulse sequence was used with a number of accumulations of 128 and a relaxation delay of 1 s.

#### 4.3.2. Inductively Coupled Plasma Atomic Emission (ICPE) Spectroscopy

ICPE analysis was performed using an ICPE-9000 spectrometer (Shimadzu, Tokyo, Japan).

The standard samples of the analyzed elements for calibration solutions were prepared from the Merck multicomponent standard in 0.1N HNO_3_ (Merck KGaA, Darmstadt, Germany). The calibration solutions were 0.001–10 mg/dm^3^. Spectral analysis of solutions was carried out in the axial mode using a mini burner with a dilution of 100 times. The calculation of the element content in the samples was made according to the calibration characteristics, taking into account the content in the blank sample. The absolute error limits were calculated based on three parallel measurements of the sample solution. The relative error in the determination of the analyte by this technique (including sample preparation and spectral analysis) was ~20%.

To determine the elemental composition of the sample, the test sample was mineralized by acid hydrolysis with microwave decomposition. The sample was transferred to a fluoroplastic beaker for microwave decomposition. The following composition of concentrated acids was used: HNO_3_ = 6 cm^3^, H_2_O_2_ = 0.5 cm^3^. The temperature control decomposition program was set for 10 min at 165 °C and 20 min at 180 °C. After cooling, the sample from fluoroplastic vessel was quantitatively transferred to a measuring flask, and a solution of 0.1N HNO_3_ was added to a mark of 10 cm^3^. The solution was transparent, and no sediment was visually detected, which indicates that the sample is suitable for analysis. The tests were performed twice, and the results averaged. The amounts of Gellan-2 and Gellan-1 were 0.1168/0.1208 g and 0.1251/0.1290 g, respectively.

#### 4.3.3. Fourier Transform Infrared (FTIR) Spectroscopy

FTIR analysis was conducted using infrared spectrophotometer (IRAffinity-1, Shimadzu, Tokyo, Japan) in the wavenumber region of 4000–400 cm^− 1^ at a resolution of 2 cm^−1^, and 32 scan signals were averaged. The samples for analysis were freeze-dried, fully mixed and ground with potassium bromide (Potassium bromide for IR spectroscopy Uvasol@), and then pressed into slices.

#### 4.3.4. Viscometery

Solution viscosity measurements were performed at 25 °C using a rolling ball microviscometer Lovis 2000 M (Anton Paar GmbH, Graz, Austria). The capillary with an inner diameter of 1.59 mm was equipped with a gold-coated steel ball (1.50 mm in diameter), and the capillary inclination angle was chosen to be 45°.

Intrinsic viscosities were determined at 25 °C by standard dilution procedures [41] via Huggins [38] and Kraemer [39] plots.

#### 4.3.5. Densitometry

Solution density measurements were performed at 25 °C using a density meter DMA 5000 M (Anton Paar GmbH, Graz, Austria). Partial specific volume was determined through density measurements according to the procedure described in [50].

#### 4.3.6. Analytical Ultracentrifugation (AUC)

Velocity sedimentation experiments were performed at 25 °C with a ProteomeLab XLI Protein Characterization System analytical ultracentrifuge (Beckman Coulter, Brea, CA, USA) using double-sector cells with aluminum centerpieces with an optical path length of 12 mm, and a four-hole analytical rotor (An-60Ti) was used. The sample and the reference sectors were loaded with 0.42 mL of studied solution and 0.44 mL of a solvent, respectively. Sedimentation profiles were obtained using the interference optical system equipped with a red laser (λ = 655 nm) as a light source. The centrifuge chamber with a loaded rotor and interferometer was vacuumed and thermostatted for at least 1.5 h before the run. The velocity sedimentation data analysis was processed using the Sedfit software (Version 16.50) (Figure S10) [54].

#### 4.3.7. Rheology

Dynamic mechanical analysis was performed using a rotational rheometer MCR 702 (Modular Compact Rheometer MCR 702e, Anton Paar, Graz, Austria) using a parallel plate (PP-50, 50 mm diameter, 1 mm gap) or coaxial cylinder geometry (CC-10) with a Peltier temperature control device. The amplitude sweep test was used to determine the linear viscoelastic region (LVR); loss and storage moduli were measured versus oscillatory strain deformation (from 0.1% up to 100%) at an angular frequency of 1 rad/s. Subsequent frequency and temperature tests were performed within the determined LVR. The temperature tests upon cooling were performed at shear strain 0.3%, frequency 1 rad/s, with a cooling rate of 3 °C/min.

### 4.4. Preparation of Gellan Gum Aqueous Solutions

Gellan gum was left to swell for 12–18 h in deionized water (Millipore Direct-Q^®^ 8 UV) (concentrations 0.6 g/dl and above) at room temperature, then placed in an ultrasonic bath at 45 °C for 2 h. Then, the solution was allowed to rest for 24 h at room temperature before further use.

### 4.5. Preparation of Gels

The gels were prepared by adding 0.2M–0.3M NaCl/KCl aqueous solution to a 0.6–1.0 g/dL aqueous solution of gellan gum in a 1:1 ratio. For all experiments, except gelation time studies, the gel was allowed to rest at least 1 h at room temperature before further use.

## Figures and Tables

**Table 1:** Cation content (wt %) of the different gellan samples used in the present work.

Sample	Ca^2+^	K^+^	Mg^2+^	Na^+^
Gellan Gum—Caramella *	0.287	2.56	0.032	0.831
Gellan-1	0.258	1.57	0.025	0.547
Gellan-2 (Gelzan™)	0.295	2.42	0.098	0.395

* Food-grade gellan gum used for purification.

**Table 2:** Concentration c (g/dL), sedimentation coefficient s_0_ (S), frictional ratio f/f_0_, and molar mass M_sf_ (kDa) calculated based on velocity sedimentation data; hydrodynamic invariant A_0_ (g cm^2^/(s^2^K mol^1/3^)) obtained for gellan in the corresponding saline solvent.

Sample	c, g/dL	s_0_, S	f/f_0_	M_sf_, kDa	A_0_·10^10^ g cm^2^/(s^2^K mol^1/3^)
Gellan-1	0.0502	1.5	8.0	100	3.0
Gellan-2	0.0503	1.6	9.4	130	2.7

**Table 3:** Formulations of gels used for DMA analysis. The gellan concentration in all cases was 0.3 g/dL.

Gel Indicator	Sample	Salt	Ion Concentration	G′_max_, Pa
G1	Gellan-1	NaCl	0.10 M	145
G2	Gellan-1	KCl	0.10 M	237
G3	Gellan-1	KCl	0.15 M	61
G4	Gellan-2	NaCl	0.10 M	195

## Data Availability

The data on the characterization and experimental results are stored at St. Petersburg State University.

## Supplementary Materials

The following supporting information can be downloaded at: https://www.mdpi.com/article/10.3390/gels11060398/s1, Figure S1: Predicted ^1^H NMR spectra of (a) acylated and (b) deacylated gellan gum; Figure S2: ^1^H NMR spectra of (a) acylated gellan gun (calculated), (b) Gellan-1 in D_2_O, and (c) food grade gellan gum used for purification in D_2_O; Figure S3: FTIR spectra of deacylated gellan (Gellan-1) and low acylated Gellan; Figure S4: The dependence of solution density (ro, g/cm^3^) on the polymer concentration, c (g/dL), for Gellan-1 (left) and Gellan-2 (right) determined for the aqueous solution at 25 °C; Figure S5: Storage (G′, squares) and loss (G″, circles) moduli as a function of time for Gellan-2 aqueous gel at 25 °C (black) and 37 °C (blue), gellan concentration is 0.3 g/dL, NaCl content is 0.1M; Figure S6: Storage (G′, squares) and loss (G″, circles) moduli as a function of time for Gellan-1 aqueous gel at 25 °C (black) and 37 °C (blue), gellan concentration is 0.3 g/dL, NaCl content is 0.1M; Figure S7: Storage (G′, squares) and loss (G″, circles) moduli as a function of time for Gellan-1 aqueous gel with 0.1M NaCl (blue) and 0.2M KCl (black) at 25 °C, gellan concentration is 0.3 g/dL; Figure S8: Amplitude Sweeps for gels G1–G4, recorded on Angular Frequency of 10 rad/s; Figure S9: Dependence of the Loss Factor (tan δ) on Shear Strain (%) for gels G1–G4; Figure S10: The normalized c_norm_(s) distributions vs. sedimentation coefficients s resolved with Sedfit at close concentrations c ~ 0.05 g/dL for Gellan-1 and Gellan-2 in corresponding water-saline solutions at 25 °C.
