# Supplementary material for: Influence of Reduced Molar Mass of Low-Acyl Gellan Gum on Weak Gel Formation and Rheological Properties
Source: Gels. 2025 May 27;11(6):398. doi: 10.3390/gels11060398 (PMC12192509; doi:10.3390/gels11060398)
Supplement: Supplementary file 1 [file gels-11-00398-s001.zip › gels-3648368-supplementary.pdf]

Supporting information

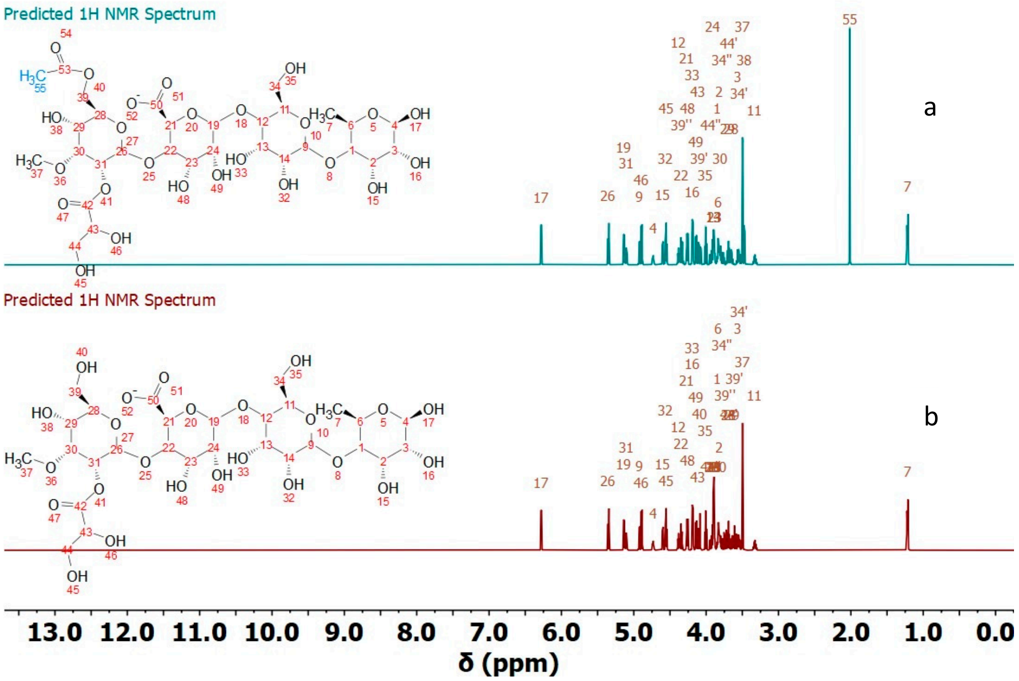

Figure S1. Predicted ^{1}H NMR spectra of (a) acylated and (b) deacylated gellan gum.

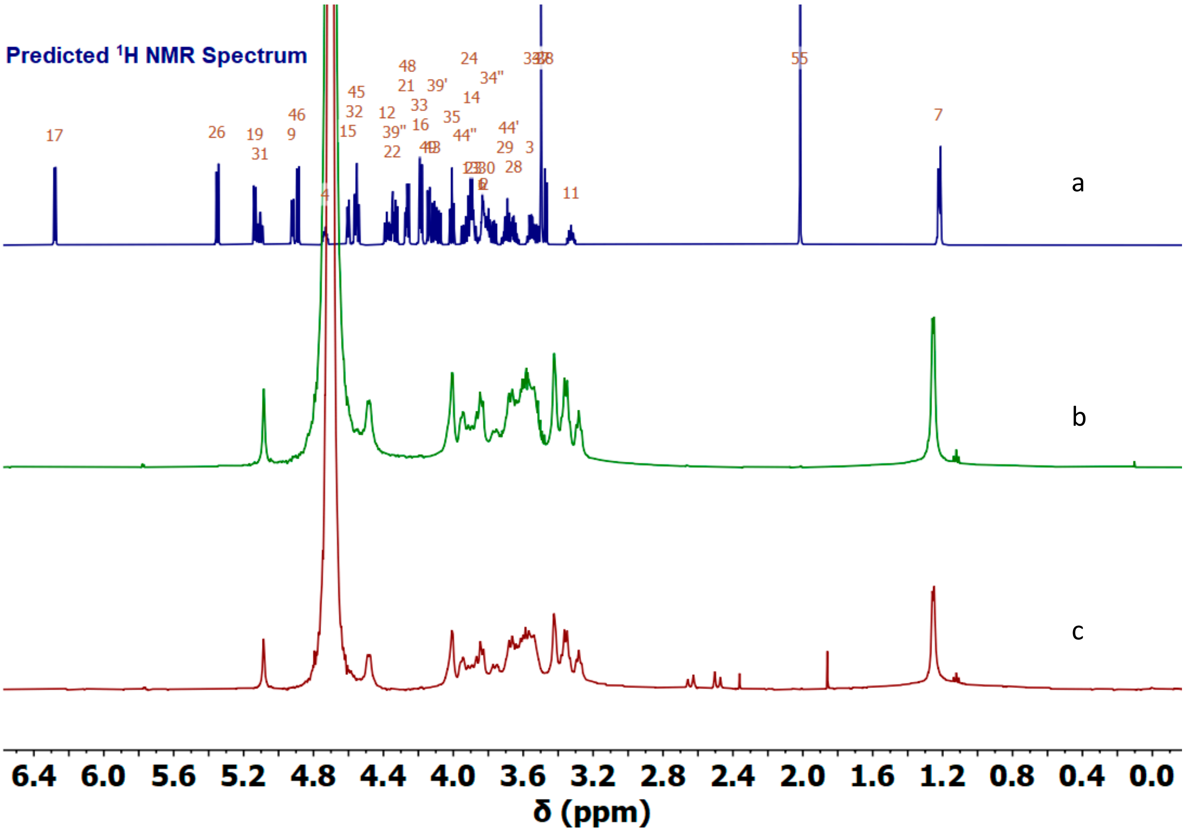

Figure S2. ^{1}H NMR spectra of (a) acylated gellan gun (calculated), (b) Gellan-1 in D_2O, and (c) food grade gellan gum used for purification in D_2O.

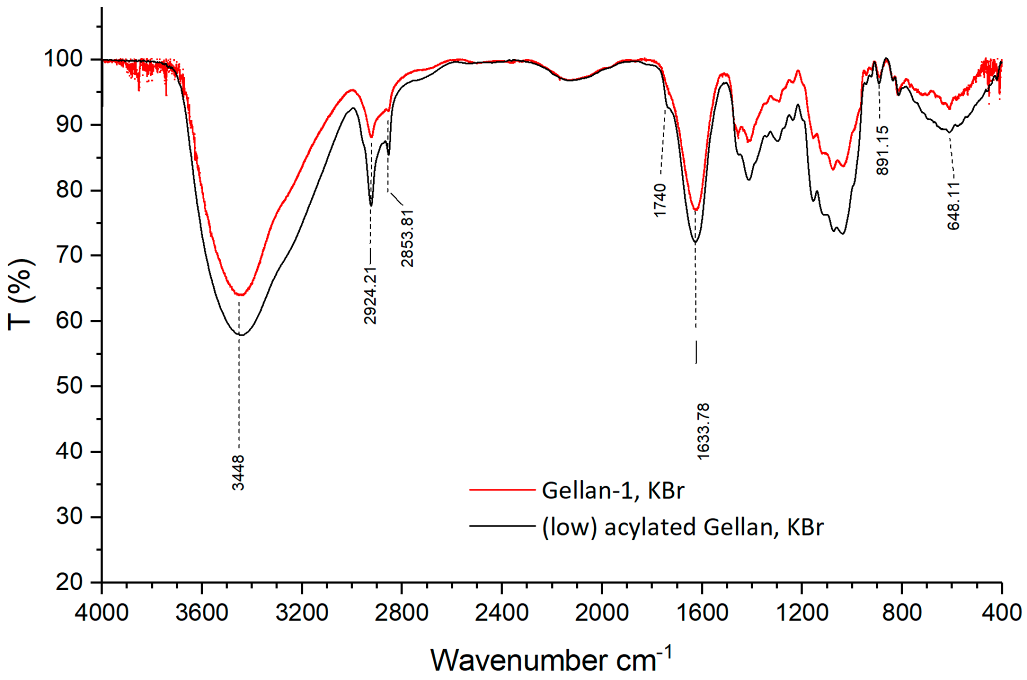

Figure S3. FTIR spectra of deacylated gellan (Gellan-1) and low acylated Gellan. The peak at 2854 cm^{-1} corresponding to CH_2 symmetric stretching band is more pronounced in gellan with acyl groups, and peak at 1740 cm^{-1}, which refers to the stretching vibration of C=O bound in the acyl groups, is present only in gellan with a certain amount of acyl groups.

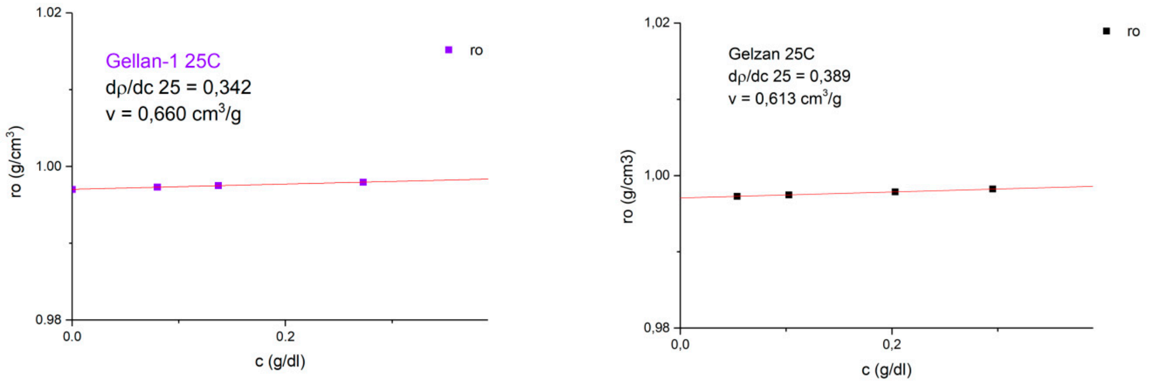

Figure S4. The dependence of solution density (ro, g/cm^3) on the polymer concentration, c (g/dL), for Gellan-1 (left) and Gellan-2 (right) determined for the aqueous solution at 25 °C.

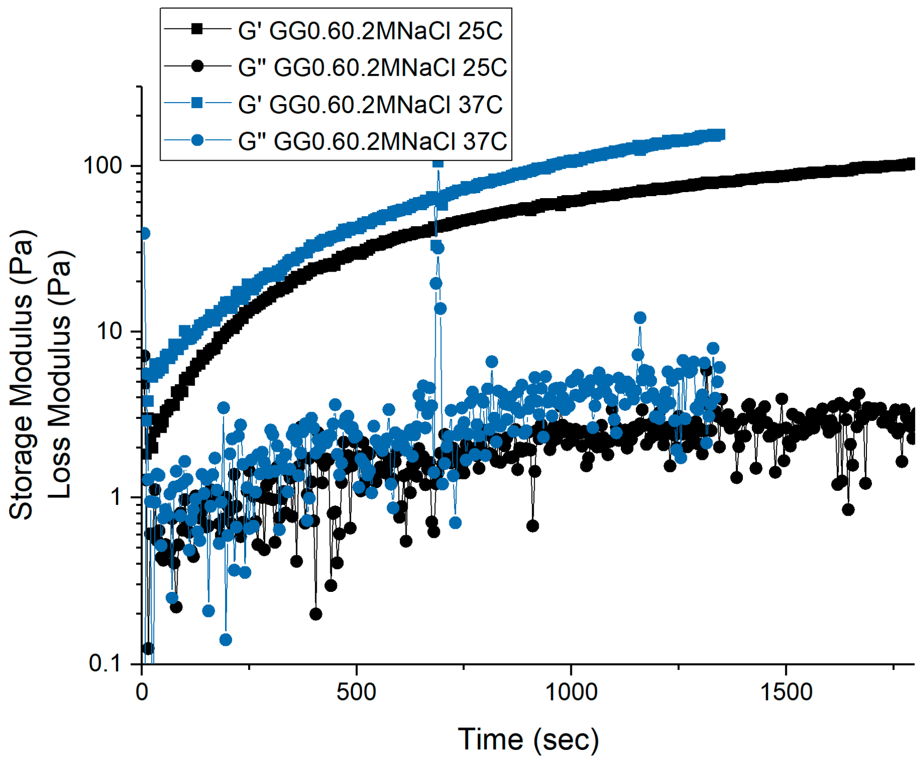

Figure S5. Storage (G', squares) and loss (G", circles) moduli as a function of time for Gellan-2 aqueous gel at 25°C (black) and 37°C (blue), gellan concentration is 0,3 g/dL, NaCl content is 0,1M.

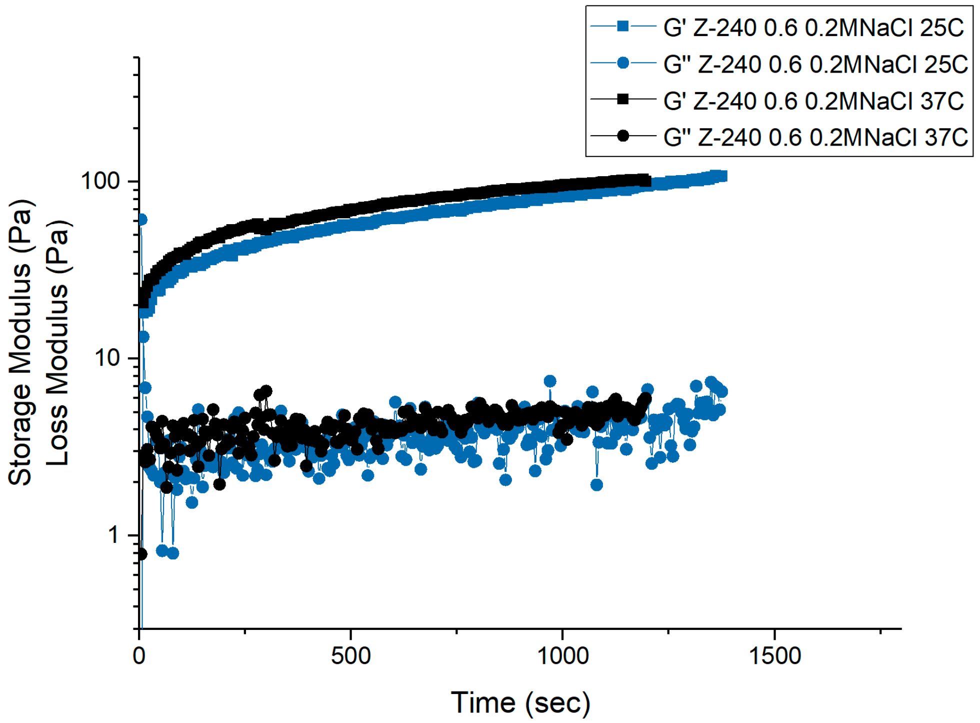

Figure S6. Storage (G', squares) and loss (G", circles) moduli as a function of time for Gellan-1 aqueous gel at 25°C (black) and 37°C (blue), gellan concentration is 0,3 g/dL, NaCl content is 0,1M.

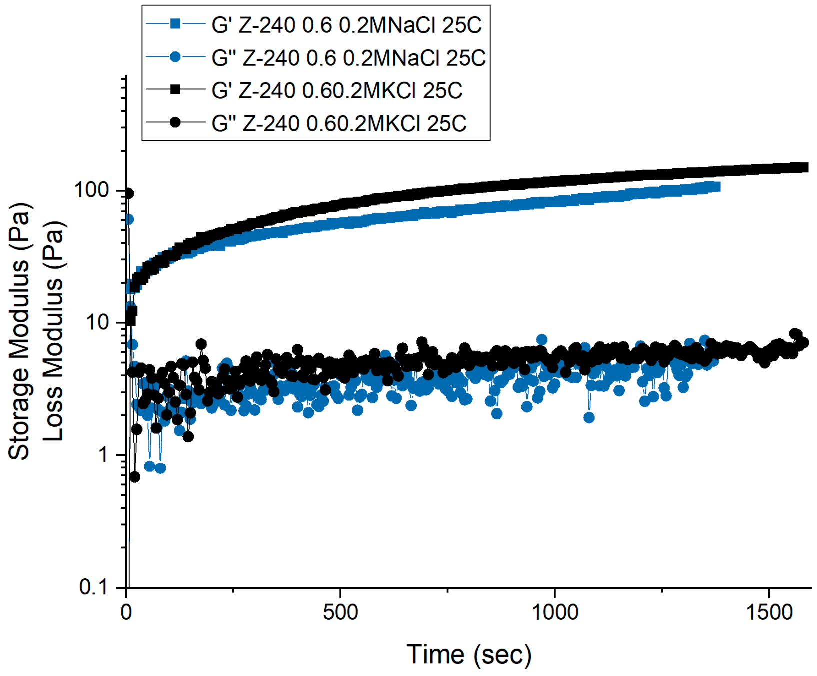

Figure S7. Storage (G', squares) and loss (G", circles) moduli as a function of time for Gellan-1 aqueous gel with 0.1M NaCl (blue) and 0.2M KCl (black) at 25°C, gellan concentration is 0,3 g/dL.

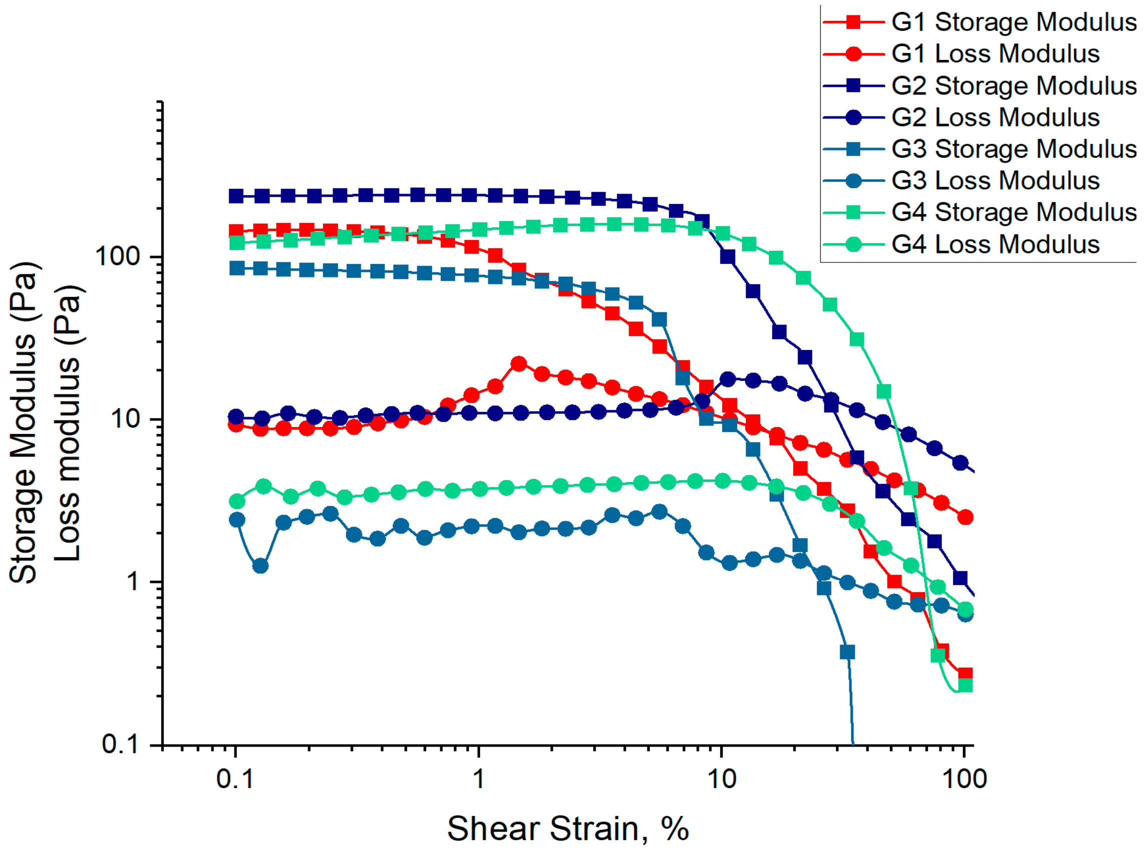

Figure S8. Amplitude Sweeps for gels G1-G4, recorded on Angular Frequency of 10 rad/s.

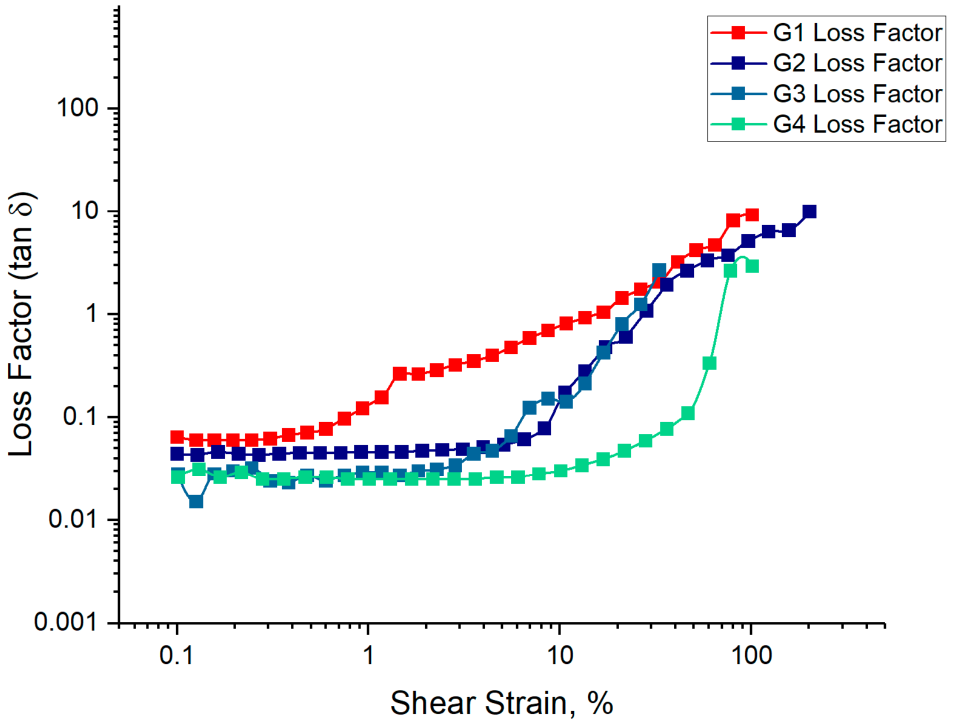

Figure S9. Dependence of the Loss Factor (tan δ) on Shear Strain (%) for gels G1-G4.

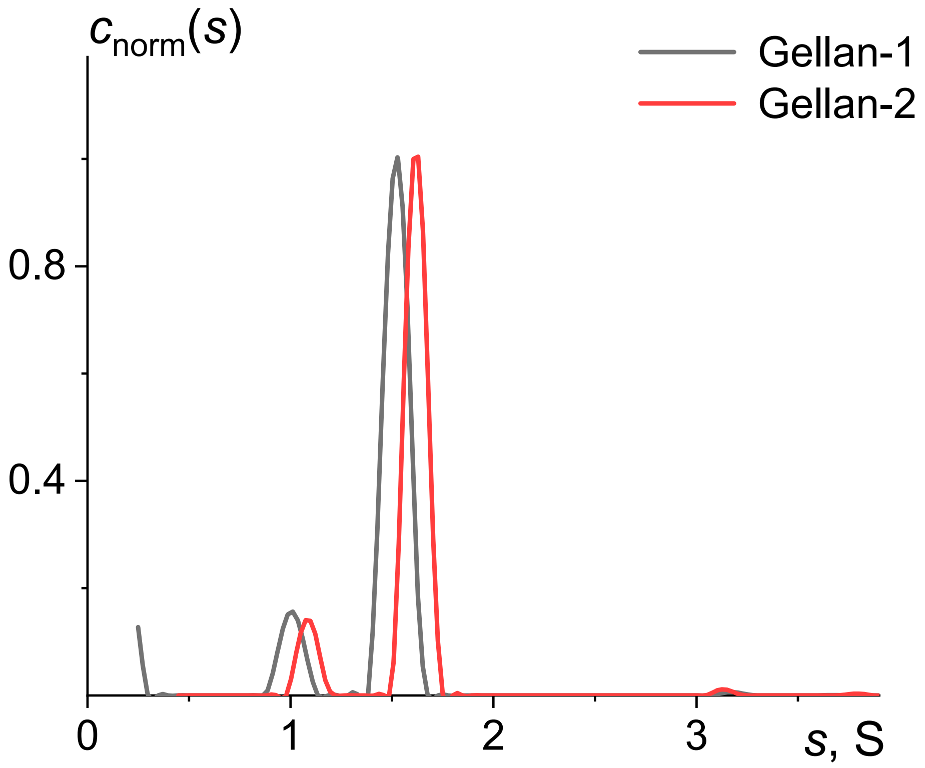

Figure S10. The normalized $c_{norm}(s)$ distributions vs. sedimentation coefficients s resolved with Sedfit at close concentrations c ~ 0.05 g/dL for Gellan-1 and Gellan-2 in corresponding water-saline solutions at 25 C.
